# The BASDAI Cut-Off for Disease Activity Corresponding to the ASDAS Scores in a Taiwanese Cohort of Ankylosing Spondylitis

**DOI:** 10.3389/fmed.2022.856654

**Published:** 2022-05-16

**Authors:** Yi-Hsing Chen, Wen-Nan Huang, Yi-Ming Chen, Kuo-Lung Lai, Tsu-Yi Hsieh, Wei-Ting Hung, Ching-Tsai Lin, Chih-Wei Tseng, Kuo-Tung Tang, Yin-Yi Chou, Yi-Da Wu, Chin-Yin Huang, Chia-Wei Hsieh, Yen-Ju Chen, Yu-Wan Liao, Hsin-Hua Chen

**Affiliations:** ^1^Division of Allergy, Immunology, and Rheumatology, Department of Internal Medicine, Taichung Veterans General Hospital, Taichung, Taiwan; ^2^School of Medicine, National Yang Ming Chiao Tung University, Taipei, Taiwan; ^3^Department of Post-Baccalaureate Medicine, College of Medicine, National Chung Hsing University, Taichung, Taiwan; ^4^Department of Business Administration, Ling-Tung University, Taichung, Taiwan; ^5^Department of Medical Research, Taichung Veterans General Hospital, Taichung, Taiwan; ^6^Department of Medical Education, Taichung Veterans General Hospital, Taichung, Taiwan; ^7^PhD Program of Business, College of Business, Feng Chia University, Taichung, Taiwan; ^8^Department of Industrial Engineering and Enterprise Information, Tunghai University, Taichung, Taiwan; ^9^Division of General Medicine, Department of Internal Medicine, Taichung Veterans General Hospital, Taichung, Taiwan; ^10^Institute of Biomedical Science and Rong-Hsing Research Center for Translational Medicine, Chung-Hsing University, Taichung, Taiwan; ^11^Institute of Public Health and Community Medicine Research Center, National Yang Ming Chiao Tung University, Taipei, Taiwan

**Keywords:** ankylosing spondylitis, biological therapy, patient-reported outcome measures, electronic medical records, BASDAI score, ASDAS

## Abstract

**Objectives:**

The Bath Ankylosing Spondylitis Disease Activity Index (BASDAI) has been widely utilized to evaluate disease activity in patients with ankylosing spondylitis (AS) by an arbitrary cut-off of ≥4 to indicate high disease activity and initiate biological therapy. The Ankylosing Spondylitis Disease Activity Score (ASDAS) is a new composite index to assess AS disease activity states that have been defined and validated. ASDAS ≥2.1 was selected as a criterion to start biological therapy. The purpose of this study was to estimate the corresponding BASDAI and ASDAS cut-off in a Taiwanese AS cohort.

**Methods:**

From November 2016 to October 2018, we assessed the ASDAS and the BASDAI regularly and recorded demographic data for 489 AS patients in Taichung Veterans General hospital (TCVGH) using an electronic patient-reported data system linked to electronic medical records. We used receiver operating characteristic curves with Youden's J statistic to determine the BASDAI values that correspond to ASDAS disease activity cut-offs (i.e., 1.3, 2.1, and 3.5).

**Results:**

In our population, the best trade-off BASDAI values corresponding to ASDAS -C-reactive protein (CRP) 1.3, 2.1, and 3.5 were 2.1, 3.1, and 3.7, respectively. The optimal BASDAI values corresponding to ASDAS-erythrocyte sedimentation rates 1.3, 2.1, and 3.5 were 2.0, 2.6, and 4.8, respectively.

**Conclusion:**

We propose a revised BASDAI cut-off based on our data, as BASDAI scores are commonly used globally. A more reasonable, lower BASDAI cut-off to initiate or change biological therapy will bring us closer to better decisions to treat AS patients.

## Introduction

To define disease activity in patients with ankylosing spondylitis (AS), the Bath Ankylosing Spondylitis Disease Activity Index (BASDAI) was introduced into practice in 1994 as a score calculated from a patient self-administered questionnaire (1). AS patients answer six questions, and their responses are scaled from 0 to 10. Patients are asked the degree of fatigue/tiredness experienced; AS-related pain in the neck, back, or hip; pain or swelling in other joints; discomfort from any areas tender to touch, pressure and discomfort from the time they wake up; and lastly, how long their morning stiffness lasts from the time they wake up. On a scale of 0 to 10, a BASDAI score ≥4 implies active disease, a consideration for initiating biologic therapy (2). Although the BASDAI is easy to administer and has been widely utilized to evaluate AS disease activity, the cut-off of BASDAI ≥4 used to indicate high disease activity in the ASAS EULAR guidelines was decided arbitrarily as a criterion to initiate biological therapy for AS patients.

The Ankylosing Spondylitis Disease Activity Score (ASDAS) computes the values of acute-phase reactants C-reactive protein (CRP) and erythrocyte sedimentation rate (ESR) in addition to the patient-reported outcomes. ASDAS, like the Disease Activity Score in rheumatoid arthritis, is a composite index that includes five items (i.e., BASDAI question 2, BASDAI question 3, BASDAI question 6, patient global assessment and laboratory data [ESR or CRP]), resulting from a formula in which each item has a different weight for the final calculation. The ASDAS, on the other hand, has well-defined cut-off values for disease activity states and has been validated. ASDAS ≥2.1 was selected as a criterion for starting biological therapy (3). In a Norwegian study, when ASDAS ≥ 2.1 and BASDAI > 4 were chosen as eligibility criteria to initiate TNF inhibitor (TNFi) treatment in AS, more patients were found eligible for TNFi using the ASDAS than the BASDAI eligibility criteria. Patients who fulfilled both criteria had the greatest likelihood of improvement, but those who fulfilled only the ASDAS criterion also improved. ASDAS could also be well applied to patient subgroups without elevated CRP and without peripheral joint swelling (4).

The construct validity of ASDAS-CRP to discriminate low and high disease activity and the cut-off values are similar in male and female patients with axSpA; however, cut-offs for ASDAS-ESR need to be defined (5). In a Turkish population study, patients with nr-axSpA and AS were assigned to low and high disease activity. The discriminatory ability of ASDAS-CRP, ASDAS-ESR, and BASDAI were found similar according to the ASAS partial remission, patient's, and physician's global assessment scores (6). A French study of 200 AS patients introduced the Patient Acceptable Symptom State (PASS) and found a significant association between disease activity and depression severity and good agreement between BASDAI and ASDAS. They found a 2.3 cut-off for both the patient-reported absence of disease activity and PASS (7). ASDAS and BASDAI showed similarly good performance in a cross-sectional setting in a local Chinese AS cohort, with ASDAS performing better in the subgroup with raised inflammatory markers (8). Several studies have used both ASDAS and BASDAI to monitor treatment responses after starting conventional synthetic disease-modifying antirheumatic drugs biological therapy. Both scores are useful to help guide clinicians in making treatment decisions in daily practice (9, 10). All of these studies are important because they correlated the ASDAS and BASDAI with physician-measured disease activity. Of note, the items of both ASDAS and BASDAI, such as fatigue, overall assessment and back pain, are nonspecific and can be found in other diseases, including fibromyalgia and degenerative joint disease. Therefore, the application of ASDAS and BASDAI in AS patients with comorbidities that shared the items of both indexes is of concern.

Determination of the BASDAI cut-off values responding to the validated ASDAS cut-off values for disease activity states is of value for clinicians to apply BASDAI to therapeutic adjustment. However, to date, only two studies have estimated the BASDAI cut-offs for disease activity states based on the validated ASDAS cut-offs (11, 12). In November 2016, we developed an electronic medical records management system for AS patients to regularly assess BASDAI, ASDAS with ESR (ASDAS-ESR) and ASDAS with CRP (ASDAS-CRP), the degree of functional impairment and in-depth evaluation of the degree of functional limitation measured by the Bath AS Functional Index, and radiographic damage measured by the modified stoke AS spinal score (mSASSS), a validated measure to assess radiographic progression (13, 14). Our electronic medical records have facilitated the study of the correlation between BASDAI and ASDAS. Therefore, the aim of the study was to determine the cut-off values of BASDAI for disease activity states corresponding to the validated ASDAS cut-offs using a retrospective analysis of a Taiwanese AS cohort from our electronic medical records.

## Methods

### Ethics Approval

This study was approved by the Institutional Review Board I & II of Taichung Veterans General Hospital (TCVGH) (Approval number: CE18321A). Any patient personal information tracked was anonymized before data analysis. Therefore, informed patient consent was not required.

### Study Design

This was a single-center retrospective, cross-sectional study.

### Data Source

To assist Rheumatologists at TCVGH, in November 2016, we had started assessing the ASDAS, the BASDAI, medications used, and clinical outcomes of all AS patients in our electronic medical record system. Our database's reliability and validity have been examined in our previous publication on the gender difference in ASAS HI among patients with AS (15). In addition to these disease-specific data, routine demographic data, comorbidities, personal history, and family history are also part of the patient records.

Using the 1984 modified New York criteria for AS, one of our 14 rheumatologists confirmed the AS diagnosis (14), and patients were registered after confirmation as TCVGH-AS cohort. Thereafter, a trained nurse used a standard questionnaire to collect information, including clinical characteristics, age at symptom onset, smoking status, history of tuberculosis, family histories, comorbidities, and extra-spinal manifestations. These questionnaires and worksheets are maintained in a standardized format to ensure reproducibility and good laboratory practices. Screening for comorbidities at presentation included hypertension, diabetes mellitus, hyperlipidemia, hepatitis B, hepatitis C, gout, coronary artery disease, stroke, periodontal disease, and osteoporosis. The extra-spinal manifestations noted at presentation were: 1. the extra-articular manifestations (EAMs) such as uveitis, psoriasis, inflammatory bowel disease; and 2. peripheral arthritis, enthesitis, and dactylitis. The rheumatologist-in-charge then confirmed the clinical characteristics. Our trained nurses assisted the AS patients with the assessments of BASDAI, ASDAS-ESR, and ASDAS-CRP. The BASFI was generated automatically into the system. An MSK-specialist radiologist reported the radiographic images of the C-spine and L-spine to calculate mSASSS. We used the data of the first assessment to compare the correlation between BASDAI and ASDAS.

### Study Subjects

From November 2016 to October 2018, a total of 489 AS patients with a complete baseline demographic and assessment data from the TCVGH electronic data system were selected. If the assessment questionnaires were incomplete for any of the four computations, BASDAI, ASDAS, BASFI, or ASAS HI, we excluded those patients from our TCVGH-AS cohort.

In general, time constraint was the only issue that prevented the completion of the questionnaire by patients. We did not collect data of patients who did not complete the questionnaires or the precise reasons for not completing the questionnaires for this study.

### Statistical Analyses

Continuous variables were reported as a mean ± standard deviation (SD), and categorical variables were reported as a percentage of patients. Differences in continuous variables were examined using the Student's *t*-test, while categorical variables were analyzed using Pearson's χ^2^ test. Spearman correlation coefficients were calculated to estimate the correlation between BASDAI and ASDAS-ESR/ASDAS-CRP because the distributions of BASDSI, ASDAS-ESR/ASDAS-CRP were not Gaussian distributions. We used receiver operating characteristic (ROC) curves with Youden's J statistic to determine the cut-off values of BASDAI that correspond to ASDAS disease activity cut-offs (i.e., 1.3, 2.1, and 3.5). We performed a statistical analysis of the ROC curve for the sensitivity of the BASDAI cut-off values in evaluating the disease states. The ROC curve analysis was the major method used to assess the correlation between two disease activities measured, as both were continuous variables. Data analysis was done using the SAS software (SAS Institute, Inc., Cary, NC, USA).

### Subgroup Analyses Based on Disease Duration

To examine whether the correlation between BASDAI and ASDAS cut-offs is consistent regardless of disease duration, we conducted a stratified analysis based on the median of disease duration (≤16 years, >16 years).

## Results

As shown in Table 1, in our patient cohort of 489 Taiwanese patients, the male to female patients' ratio was 3:1. The mean age at presentation was 44.2 years, with an SD of 13.8 years. On average, patients had AS-related symptoms for 18 years. The average age at which AS was diagnosed was 29 years for women and 25 years for men. While one-third of the men were smokers, only 7% of the women reported a smoking history. The presence of comorbidities such as hypertension, diabetes, hyperlipidemia, hepatitis B, hepatitis C, chronic renal failure, gout, coronary artery disease, stroke, periodontal disease, and osteoporosis in the male and female cohorts is shown in Table 1. As shown in Table 1, almost one-third of the patient population (31.1%) used biologics. The non-biologic therapies used included methotrexate (6.7%), sulfasalazine (40.7%), NSAIDs (88.8%), tramadol (26%) and corticosteroids (11.9%). Table 2 revealed the baseline disease activity measures of the study population. Of the 489 AS patients, 179 (36.6%) had an ASDAS-CRP ≥2.1, and 25 (5.1%) of the study population had very high disease activity.

**Table 1:** Demographic data and clinical characteristics at presentation of patients with AS.

	**Total**	**Female**	**Male**	
	***n* = 489 (100%)**	***n* = 114 (23.3%)**	***n* = 375 (76.7%)**	***P*-value**
**Age, years (Mean±SD)**	44.2 ± 13.8	42.9 ± 13.8	44.6 ± 13.8	0.253
**Age at AS diagnosis, years (mean** **±SD)**	25.9 ± 10.8	29.2 ± 12.2	25.0 ± 10.2	0.001
**Symptom duration, year (mean** **±SD)**	18.3 ± 11.9	13.7 ± 10.4	19.6 ± 12.1	<0.001
**HLA-B27**	441 (90.4)	93 (81.6)	348 (93)	<0.001
**Smoke**	164 (33.5)	7 (6.1)	157 (41.9)	<0.001
**Comorbidities**				
Hypertension	99 (20.2)	12 (10.5)	87 (23.2)	0.003
Diabetes mellitus	35 (7.2)	5 (4.4)	30 (8.0)	0.190
Hyperlipidemia	71 (14.5)	9 (8.0)	62 (16.5)	0.024
Hepatitis B	59 (12.1)	11 (9.6)	48 (12.8)	0.366
Hepatitis C	12 (2.5)	3 (2.6)	9 (2.4)	0.889
Chronic renal failure	15 (3.1)	2 (1.8)	13 (3.5)	0.353
Gout	23 (4.7)	2 (1.8)	21 (5.6)	0.089
Coronary artery disease	17 (3.5)	1 (0.9)	16 (4.3)	0.139^*^
Stroke	2 (0.4)	0 (0.0)	2 (0.5)	1.000
Periodontal disease	112 (22.9)	12 (10.5)	100 (26.7)	<0.001
Osteoporosis	35 (7.2)	10 (8.8)	25 (6.7)	0.430
**Extra-spinal manifestation**				
Uveitis	135 (27.6)	32 (28.1)	103 (27.5)	0.900
Psoriasis	37 (7.6)	6 (5.3)	31 (8.3)	0.285
Crohn's disease	0 (0.0)	0 (0.0)	0 (0.0)	-
Ulcerative colitis	2 (0.4)	1 (0.9)	1 (0.3)	0.371^*^
Peripheral arthritis	114 (23.4)	29 (25.4)	85 (22.8)	0.559
Enthesitis	75 (15.3)	18 (15.8)	57 (15.2)	0.878
Dactylitis	12 (2.5)	0 (0.0)	12 (3.2)	0.077^*^
**Past history**				
Total hip replacement	20 (4.1)	1 (0.9)	19 (5.1)	0.057^*^
Total knee replacement	2 (0.4)	1 (0.9)	1 (0.3)	0.412^*^
Fracture	49 (10.0)	8 (7.0)	41 (10.9)	0.223
Tuberculosis	16 (3.3)	2 (1.8)	14 (3.7)	0.382^*^
Palindromic rheumatism	5 (1.0)	0 (0.0)	5 (1.3)	0.595^*^
Family history of AS (first or second-degree relatives)	193 (39.5)	50 (43.9)	143 (38.1)	0.273
First degree relatives	93 (19.9)	22 (20.2)	71 (19.8)	0.926
Second-degree relatives	137 (28.8)	37 (33.0)	100 (27.5)	0.262
**Current medications**				
Biologics	152 (31.1)	40 (35.1)	112 (29.9)	0.292
Methotrexate	33 (6.7)	9 (7.9)	24 (6.4)	0.577
Sulfasalazine	199 (40.7)	41 (36.0)	158 (42.1)	0.240
NSAID	434 (88.8)	102 (89.5)	332 (88.5)	0.781
Tramadol/acetaminophen	127 (26.0)	28 (24.6)	99 (26.4)	0.695
Corticosteroid	58 (11.9)	14 (12.3)	44 (11.7)	0.849

*Data were shown as number (percentage) unless specified otherwise*.

* *Fisher's exact test*.

*AS, ankylosing spondylitis; HLA, human leukocyte antigen; NSAID, non-steroidal anti-inflammatory drug; SD, standard deviation*.

**Table 2:** Baseline disease activity measures of the study population.

**Disease activity measures**	***n* = 489**
BASDAI Q1 (fatigue), median (IQR)	3.0 (2.0–5.0)
BASDAI Q2 (neck/back/hip pain), median (IQR)	3.0 (2.0–5.0)
BASDAI Q3 (peripheral joint pain/swelling), median (IQR)	1.0 (0.0–3.0)
BASDAI Q4 (enthesitis), median (IQR)	1.0 (0.0–3.0)
BASDAI Q5 (severity of morning stiffness), median (IQR)	3.0 (1.0–4.0)
BASDAI Q6 (duration of morning stiffness), median (IQR)	2.0 (1.0–3.0)
PGA, median (IQR)	3.0 (1.0–4.0)
CRP, median (IQR)	0.3 (0.1–0.6)
ESR, median (IQR)	8.0 (3.0–15.0)
BASDAI, median (IQR)	2.4 (1.4–3.7)
ASDAS-CRP, median (IQR)	1.8 (1.2–2.3)
Inactive disease, *n* (%)	130 (26.6)
Moderate disease activity, *n* (%)	180 (36.8)
High disease activity, *n* (%)	154 (31.5)
Very high disease activity, *n* (%)	25 (5.1)
ASDAS-ESR, median (IQR)	1.7 (1.2–2.3)
Inactive disease, *n* (%)	134 (27.4)
Moderate disease activity, *n* (%)	184 (37.6)
High disease activity, *n* (%)	149 (30.5)
Very high disease activity, *n* (%)	22 (4.5)

*BASDAI, Bath Ankylosing Spondylitis Disease Activity Index; Q, question; IQR, interquartile range; PGA, patient global assessment; CRP, C-reactive protein; ESR, erythrocyte sedimentation rate; ASDAS, Ankylosing Spondylitis Disease Activity Score*.

BASDAI was significantly correlated with ASDAS-CRP (r = 0.7203, *p* < 0.0001) and ASDAS-ESR (r = 0.7590, *p* < 0.0001). As shown in Table 3, the best cut-off BASDAI values corresponding to ASDAS-CRP 1.3, 2.1, and 3.5 were 2.1, 3.1, and 3.7, respectively. The inactive disease was found in 42.9% of patients, who had an ASDAS- CRP value <1.3, corresponding with a BASDAI cut-off value of 2.1. Low disease activity was found in 35% of patients, marked by an ASDAS-CRP value of 1.3 to 2.1, corresponding to a BASDAI cut-off value of 2.1–3.1. High disease activity was found in 18% patients, marked by an ASDAS-CRP value of 2.1–3.5, corresponding to a BASDAI cut-off value of 3.1–3.7. Very high disease activity was found in 4.1% patients, marked by ASDAS-CRP values of 3.5, corresponding to a BASDAI cut-off value of 3.7. Notably, the optimal BASDAI values corresponding to ASDAS-ESR 1.3, 2.1, and 3.5 were 2.0, 2.6, and 4.8, respectively.

**Table 3:** Optimal BASDAI cut-off values corresponding to ASDAS cut-offs using ROC curve with Youden's J statistic in AS patients.

	**BASDAI cut-off**	**AUC (95% CI)**	**Specificity (95% CI)**	**Specificity (95% CI)**	**PPV (95% CI)**	**NPV (95% CI)**
ASDAS-CRP 1.3	2.1	0.76 (0.72–0.80)	0.70 (0.65–0.75)	0.82 (0.75–0.88)	0.92 (0.88–0.95)	0.50 (0.43–0.57)
ASDAS-CRP 2.1	3.1	0.78 (0.74–0.82)	0.69 (0.62–0.76)	0.87 (0.83–0.91)	0.76 (0.68–0.82)	0.83 (0.79–0.87)
ASDAS-CRP 3.5	3.7	0.82 (0.75–0.89)	0.84 (0.66–0.95)	0.81 (0.77–0.84)	0.23 (0.15–0.31)	0.99 (0.97–1.00)
ASDAS-ESR 1.3	2.0	0.80 (0.77–0.84)	0.74 (0.69–0.78)	0.87 (0.80–0.92)	0.94 (0.90–0.96)	0.56 (0.48–0.62)
ASDAS-ESR 2.1	2.6	0.79 (0.76–0.83)	0.81 (0.74–0.86)	0.78 (0.73–0.82)	0.66 (0.59–0.73)	0.88 (0.84–0.92)
ASDAS-ESR 3.5	4.8	0.79 (0.70–0.89)	0.67 (0.46–0.83)	0.92 (0.89–0.94)	0.33 (0.21–0.47)	0.98 (0.96–0.99)

*ASADS, ankylosing spondylitis disease activity score; AS, ankylosing spondylitis; AUC, area under the curve; BASDAI, bath ankylosing spondylitis disease activity index; CI, confidence interval; CRP, C-reactive protein; ESR, erythrocyte sedimentation rate; ROC, receiver operating characteristic; PPV, positive predictive value; NPV, negative predictive value*.

**Figure 1:**
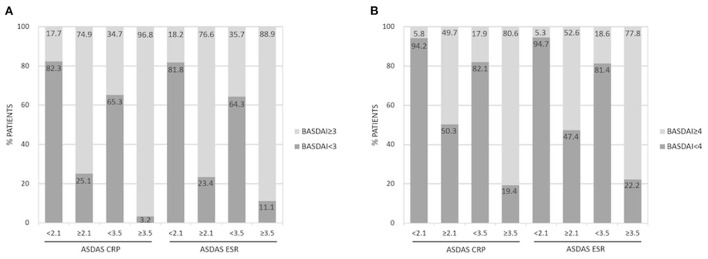
Agreement between high and very high disease activity states according to ASDAS-CRP/ASDAS-ESR and BASDAI ≥3 **(A)** and BASDAI ≥4 **(B)**.

Tables 4, 5 reveal the degree of agreement of disease activity states based on the corresponding BASDAI cut-off values with those based on ASDAS-CRP and ASDAS-ESR cut-off values, respectively. Figure 1 and Supplementary Table 1 show the agreement between BASDAI ≥3, ≥4 and high and very high disease activity states according to ASDAS values.

**Table 4:** Degree of agreement between disease activity states based on BASDAI and ASDAS-CRP cut-off values.

	**ASDAS-CRP <1.3**	**1.3 ≤ASDAS-CRP <2.1**	**2.1 ≤ASDAS-CRP ≤3.5**	**ASDAS-CRP > 3.5**
	***n* = 130**	***n* = 180**	***n* = 154**	***n* = 25**
BASDAI <2.1	107 (82.3)	83 (46.1)	19 (12.3)	0 (0.0)
2.1 ≤ BASDAI <3.1	19 (14.6)	57 (31.7)	30 (19.5)	0 (0.0)
3.1 ≤ BASDAI ≤ 3.7	2 (1.5)	22 (12.2)	33 (21.4)	2 (8.0)
BASDAI > 3.7	2 (1.5)	18 (10.0)	72 (46.8)	23 (92.0)

*BASDAI, Bath Ankylosing Spondylitis Disease Activity Index; ASDAS, Ankylosing Spondylitis Disease Activity Score; CRP, C-reactive protein*.

*Weighted kappa: 0.464, p < 0.001*.

**Table 5:** Degree of agreement between disease activity states based on BASDAI and ASDAS-ESR cut-off values.

	**ASDAS-ESR <1.3**	**1.3 ≤ASDAS-ESR <2.1**	**2.1 ≤ASDAS-ESR ≤3.5**	**ASDAS-ESR > 3.5**
	***n* = 134**	***n* = 184**	***n* = 149**	***n* = 22**
BASDAI <2.0	113 (84.3)	64 (34.8)	14 (9.4)	0 (0.0)
2.0 ≤ BASDAI <2.6	13 (9.7)	51 (27.7)	12 (8.1)	0 (0.0)
2.6 ≤ BASDAI ≤ 4.8	8 (6.0)	67 (36.4)	87 (58.4)	5 (22.7)
BASDAI > 4.8	0 (0.0)	2 (1.1)	36 (24.2)	17 (77.3)

*BASDAI, Bath Ankylosing Spondylitis Disease Activity Index; ASDAS, Ankylosing Spondylitis Disease Activity Score; ESR, erythrocyte sedimentation rate*.

*Weighted kappa: 0.538, p < 0.001*.

As shown in Supplementary Tables 2, 3, the corresponding cut-off values of BASDAI in patients with a symptom duration > 16 years were larger than those in patients with a symptom duration ≤ 16 years. The mean ± standard deviation mSASSS was higher in AS patients with a symptom duration > 16 years than that in patients with a symptom duration of ≤ 16 years (24.19 ± 24.24 vs. 10.73 ± 16.43, *p* < 0.001).

## Discussion

The correlation coefficients between the BASDAI and ASDAS-CRP/ASDAS-ESR were 0.7203/0.7590 (*p* < 0.0001, both), which were consistent with those reported in prior studies (7, 16–18). Our data determined the disease activity state cut-offs for BASDAI at 2.1/2.0, 3.1/2.6, and 3.7/4.8 for inactive disease, low disease activity, high disease activity, and very high disease activity, while using ASDAS-CRP/ASCAS-ESR as references. These cut-offs are much lower than the cut-offs of BASDAI currently used to define disease states, which are at 3, 4, and 6, respectively (3). Although the corresponding BASDAI cut-off values seem to be larger in AS patients with a symptom duration > 16 years compared with those aged ≤16 years, they were still lower than the currently used BASDAI cut-offs. The higher corresponding cut-off values of BASDAI in patients with longer disease duration may be explained by greater structure damage in the spine. Given that the BASDAI cut-offs at 3, 4, 6 for disease activity states have not been validated, our estimated BASDAI cut-offs can be used as options to define AS disease activity states in real-world practice when ASDAS are not available.

It is difficult to define a state of remission in AS patients. Godfrin-Valnet M et al. found that the cut-off of ASDAS corresponding to the inactive disease based on patient-reported disease activity was 2.3 (7). However, in longitudinal studies, a significant proportion of AS patients with an ASDAS value of a1.3–2.1 still suffered from radiographic progression (19, 20). Therefore, ASDAS <1.3 is suggested to be the global therapeutic target considering long-term consequences (21). Our first finding that inactive disease, set at an ASDAS-CRP/ASDAS-ESR value <1.3, corresponding with a BASDAI cut-off value of 2.1/2.0, suggests that the treatment goals of remission/inactive disease might be set at BASDAI 2.1 or 2.0, as opposed to the traditional goals of BASDAI 3 values.

In patients with AS, ASDAS-CRP ≥2.1 and BASDAI ≥4 have been widely used to suggest a change of therapy, such as initiating biologics (3, 22). However, ASDAS-CRP ≥2.1 can take more patients eligible to initiate biologics than BASDAI ≥4 (2, 23, 24). Our second finding that high/very high disease activity, marked by an ASDAS-CRP value of ≥ 2.1, corresponds to a BASDAI cut-off value of 3.1, suggesting an inadequate response to prior therapy and a need to adjust treatment (3, 22, 25). In a Korean cohort of 333 patients with axial spondyloarthritis, Chan et al. found that the BASDAI values corresponding to ASDAS-CRP values of 1.3, 2.1 and 3.5 were 1.9, 3.5 and 4.9, respectively (11). Their defined BASDAI cut-off value for the inactive disease was consistent with our finding. However, their estimated BASDAI cut-offs for high disease activity and very high disease activity were higher than our findings. In a Turkish cohort of 396 patients with AS, the estimated BASDAI cut-off values corresponding to ASDAS-CRP values of 2.1 and 3.5 were 2.4 and 3.7, respectively (12). The data of this Turkish cohort were more consistent with our findings than those of the Korean cohort. Possible explanations of the discrepancies in the Korean cohort were the exclusion of fibromyalgia, the inclusion of patients with non-radiographic axial spondyloarthritis and the inclusions of patients with a shorter symptom duration than our cohort. However, we also conducted a subgroup analysis based on the median value of symptom duration and found consistent results. Of note, ASDAS-ESR data were only available in our cohort and cannot be compared with the data of other studies.

One limitation of our study is that this is a single-center analysis in a Taiwanese cohort. The second limitation is that our patient population was enrolled as per convenience, which means no specific exclusion criteria have been used. The third limitation is that a close differential diagnosis of fibromyalgia, which could influence our statistical correlation analysis, was not screened in this cohort. Fourth, the long symptom duration and the small proportion of very high disease activity of our cohort limited the application of our findings on AS patients with short disease duration and very high disease activity. Finally, the findings in our Taiwanese cohort may not be generalized to other populations.

## Conclusion

The cut-offs used by rheumatologists for escalating therapy is currently based on the 2016 ASAS EULAR management recommendations for axial spondyloarthritis at ASDAS ≥2.1 vs. BASDAI ≥4. We propose a revision to a lower BASDAI cut-off based on our data, as BASDAI scores are more commonly used globally. A more reasonable and accurate, lower BASDAI cut-off to initiate or change biological therapy brings us one step closer to better decisions to treat AS patients with bDMARDs, ultimately reducing the disability due to progression in AS patients. In addition to the early initiation of therapy with highly effective treatments, long-term follow-up data on treatment outcomes with different agents can guide the selection of these agents. Further studies are warranted to evaluate the long-term outcomes of AS patients with various disease activity states based on the BASDAI cut-offs corresponding to the ASDAS cut-offs.

We must find the most accurate markers to predict prognosis, severe disease, and treatment response. For the next steps, our large, broad-based foundation cohort of AS patients is a great resource when complemented with more detailed follow-up information in the longer term to achieve treatment goals of slowing down AS disease progression.

## Data Availability Statement

The raw data supporting the conclusions of this article will be made available by the authors, without undue reservation.

## Ethics Statement

The studies involving human participants were reviewed and approved by the Institutional Review Board I of Taichung Veterans General Hospital (Approval number: CE18321A). The Ethics Committee waived the requirement of written informed consent for participation.

## Author Contributions

H-HC, Y-MC, and K-LL: conceived and designed the experiments. T-YH, W-TH, C-TL, C-WT, K-TT, Y-YC, Y-DW, C-WH, Y-JC, and H-HC: acquired data. Y-MC, K-LL, C-YH, and H-HC: contributed materials/analysis tools. H-HC, Y-HC, W-NH, Y-MC, and K-LL: wrote the article. All authors contributed to the article and approved the submitted version.

## Funding

This study received funding from Novartis Pharma AG. The funder was not involved in the study design, collection, analysis, interpretation of data, the writing of this article or the decision to submit it for publication.

## Conflict of Interest

The authors declare that the research was conducted in the absence of any commercial or financial relationships that could be construed as a potential conflict of interest.

## Publisher's Note

All claims expressed in this article are solely those of the authors and do not necessarily represent those of their affiliated organizations, or those of the publisher, the editors and the reviewers. Any product that may be evaluated in this article, or claim that may be made by its manufacturer, is not guaranteed or endorsed by the publisher.
