# Supplementary material for: The BASDAI Cut-Off for Disease Activity Corresponding to the ASDAS Scores in a Taiwanese Cohort of Ankylosing Spondylitis
Source: Front Med (Lausanne). 2022 May 16;9:856654. doi: 10.3389/fmed.2022.856654 (PMC9149077; doi:10.3389/fmed.2022.856654)
Supplement: Supplementary file 1 [file Table_1.DOCX]

**The BASDAI cut-off for disease activity corresponding to the ASDAS scores in a Taiwanese cohort of ankylosing spondylitis**

**Yi-Hsing Chen^*^, Wen-Nan Huang^*^, Yi-Ming Chen^*^, Kuo-Lung Lai^*^, Tsu-Yi Hsieh, Wei-Ting Hung, Ching-Tsai Lin, Chih-Wei Tseng^1,3^, Kuo-Tung Tang, Yin-Yi Chou, Yi-Da Wu, Chin-Yin Huang, Chia-Wei Hsieh, Yen-Ju Chen, Yu-Wan Liao^1,3^, Hsin-Hua Chen^#^**

**Supplemental materials**

**Table A. Agreement between BASDAI≥3, ≥4 and high and very high disease activity states according to ASDAS**

|  | **ASDAS_CRP** | | | | **ASDAS_ESR** | | | | **Total** |
| --- | --- | --- | --- | --- | --- | --- | --- | --- | --- |
|  | **<2.1** | **≥2.1** | **<3.5** | **≥3.5** | **<2.1** | **≥2.1** | **<3.5** | **≥3.5** |  |
| BASDAI |  |  |  |  |  |  |  |  |  |
| <3 | 255 | 45 | 299 | 1 | 260 | 40 | 297 | 3 | 300 |
| ≥3 | 55 | 134 | 159 | 30 | 58 | 131 | 165 | 24 | 189 |
| <4 | 292 | 90 | 376 | 6 | 301 | 81 | 376 | 6 | 382 |
| ≥4 | 18 | 89 | 82 | 25 | 17 | 90 | 86 | 21 | 107 |
| Total | 310 | 179 | 458 | 31 | 318 | 171 | 462 | 27 | 489 |

ASADS, ankylosing spondylitis disease activity score; BASDAI, bath ankylosing spondylitis disease activity index, CRP, C-reactive protein; ESR, erythrocyte sedimentation rate.

**Table B**. **Optimal BASDAI cut-off values corresponding to ASDAS cut-offs using ROC curve with Youden’s J statistic in AS patients with a symptom duration ≤ 16 years**

|  | **BASDAI cut-off** | **AUC (95% CI)** | **Specificity (95% CI)** | **Specificity (95% CI)** | **PPV (95% CI)** | **NPV (95% CI)** |
| --- | --- | --- | --- | --- | --- | --- |
| ASDAS-CRP 1.3 | 2.1 | 0.78 (0.73–0.84) | 0.71 (0.63–0.77) | 0.86 (0.76–0.93) | 0.93 (0.87–0.96) | 0.55 (0.45–0.64) |
| ASDAS-CRP 2.1 | 2.5 | 0.80 (0.75–0.85) | 0.82 (0.73–0.90) | 0.78 (0.71–0.84) | 0.66 (0.56–0.75) | 0.90 (0.83–0.94) |
| ASDAS-CRP 3.5 | 3.7 | 0.84 (0.76–0.93) | 0.84 (0.60–0.97) | 0.84 (0.79–0.89) | 0.31 (0.19–0.45) | 0.98 (0.96–1.00) |
| ASDAS-ESR 1.3 | 1.7 | 0.84 (0.79–0.89) | 0.82 (0.76–0.88) | 0.85 (0.75–0.93) | 0.94 (0.89–0.97) | 0.64 (0.54–0.74) |
| ASDAS-ESR 2.1 | 2.6 | 0.80 (0.75–0.86) | 0.79 (0.69–0.87) | 0.82 (0.75–0.88) | 0.71 (0.61–0.79) | 0.87 (0.81–0.92) |
| ASDAS-ESR 3.5 | 2.7 | 0.80 (0.74–0.87) | 0.94 (0.73–1.00) | 0.66 (0.60–0.72) | 0.18 (0.11–0.27) | 0.99 (0.96–1.00) |

Abbreviations: ASADS, ankylosing spondylitis disease activity score; AS, ankylosing spondylitis; AUC, area under the curve; BASDAI, bath ankylosing spondylitis disease activity index; CI, confidence interval; CRP, C-reactive protein; ESR, erythrocyte sedimentation rate; ROC, receiver operating characteristic; PPV, positive predictive value; NPV, negative predictive value.

**Table C**. **Optimal BASDAI cut-off values corresponding to ASDAS cut-offs using ROC curve with Youden’s J statistic in AS patients with a symptom duration > 16 years**

|  | **BASDAI cut-off** | **AUC (95% CI)** | **Specificity (95% CI)** | **Specificity (95% CI)** | **PPV (95% CI)** | **NPV (95% CI)** |
| --- | --- | --- | --- | --- | --- | --- |
| ASDAS-CRP 1.3 | 2.4 | 0.73 (0.67–0.79) | 0.60 (0.53–0.68) | 0.86 (0.74–0.94) | 0.93 (0.87–0.97) | 0.41 (0.32–0.49) |
| ASDAS-CRP 2.1 | 2.8 | 0.77 (0.72–0.83) | 0.76 (0.66–0.84) | 0.79 (0.71–0.85) | 0.70 (0.60–0.78) | 0.83 (0.76–0.89) |
| ASDAS-CRP 3.5 | 3.2 | 0.80 (0.71–0.89) | 0.92 (0.62–1.00) | 0.69 (0.62–0.75) | 0.13 (0.07–0.23) | 0.99 (0.97–1.00) |
| ASDAS-ESR 1.3 | 2.3 | 0.80 (0.75–0.85) | 0.69 (0.61–0.76) | 0.91 (0.81–0.97) | 0.95 (0.90–0.98) | 0.53 (0.43–0.62) |
| ASDAS-ESR 2.1 | 3.3 | 0.80 (0.74–0.85) | 0.73 (0.62–0.82) | 0.87 (0.80–0.92) | 0.74 (0.63–0.83) | 0.86 (0.80–0.91) |
| ASDAS-ESR 3.5 | 4.4 | 0.76 (0.59–0.92) | 0.67 (0.30–0.93) | 0.85 (0.80–0.90) | 0.15 (0.06–0.30) | 0.98 (0.96–1.00) |

Abbreviations: ASADS, ankylosing spondylitis disease activity score; AS, ankylosing spondylitis; AUC, area under the curve; BASDAI, bath ankylosing spondylitis disease activity index; CI, confidence interval; CRP, C-reactive protein; ESR, erythrocyte sedimentation rate; ROC, receiver operating characteristic; PPV, positive predictive value; NPV, negative predictive value.
